# HIV-1 tuberculosis-associated immune reconstitution inflammatory syndrome

**DOI:** 10.1007/s00281-015-0532-2

**Published:** 2015-09-30

**Authors:** Rachel P. J. Lai, Graeme Meintjes, Robert J. Wilkinson

**Affiliations:** The Francis Crick Institute, Mill Hill Laboratory, London, NW7 1AA UK; Institute of Infectious Disease and Molecular Medicine, Faculty of Health Sciences, University of Cape Town, Room 3.03.05, Wolfson Pavilion, Anzio Road, Observatory, Cape Town, 7925 South Africa; Department of Medicine, Imperial College London, London, W2 1PG UK; Department of Medicine, University of Cape Town, Observatory, Cape Town, 7925 South Africa

**Keywords:** HIV-1 infection, Tuberculosis, Immune reconstitution inflammatory syndrome, Drug therapy-complications

## Abstract

Patients co-infected with HIV-1 and tuberculosis (TB) are at risk of developing TB-associated immune reconstitution inflammatory syndrome (TB-IRIS) following commencement of antiretroviral therapy (ART). TB-IRIS is characterized by transient but severe localized or systemic inflammatory reactions against *Mycobacterium tuberculosis* antigens. Here, we review the risk factors and clinical management of TB-IRIS, as well as the roles played by different aspects of the immune response in contributing to TB-IRIS pathogenesis.

## HIV-tuberculosis co-infection

Human immunodeficiency virus type 1 (HIV-1) and *Mycobacterium tuberculosis* are two major infectious diseases and each was responsible for causing approximately 1.5 million deaths in 2013 [1, 2]. There were an estimated 9.0 million cases of active tuberculosis (TB) in 2013, among which about 1.1 million (13 %) were HIV-1 co-infected [2]. The prevalence of HIV-1 co-infection among TB cases is the highest in Africa, accounting for around 78 % of the total HIV-TB cases.

HIV-1 infection and active TB disease individually lead to severe immune impairment and they can also exacerbate the disease progression and pathology of one another. Tuberculosis can cause lymphocytopenia in the absence of HIV-1 or other cause of immunodeficiency [3, 4]. When CD4^+^ T cells counts were controlled for, HIV-1 patients with active TB disease still have more a rapid course of disease progression and poorer survival rate than those without active TB [5]. Furthermore, people living with HIV-1 are 26–31 times more likely to develop TB than HIV-uninfected individuals [6].

HIV-1 causes functional disruption of the immune response and impairs the host’s ability to control *M. tuberculosis* infection via several mechanisms (reviewed in [7]). Firstly, HIV-1 replication is higher in stimulated macrophages at the site of *M. tuberculosis* infection [8, 9], and higher viral loads in bronchoalveolar lavage and in pleural fluid have been observed [10, 11]. Secondly, HIV-1-infected macrophages have reduced secretion of TNF-α that associates with reduced apoptosis in response to *M. tuberculosis* [12, 13]. While expression of TLR2 and TLR4 are preserved in HIV-1-infected macrophages, TNF-α secretion in response to specific TLR agonists, as well as signal transduction through IRAK-1 and NF-κB nuclear translocation, are significantly reduced [13]. Finally, HIV-1 infects and depletes CD4^+^ T cells, including those that are *M. tuberculosis*-specific, resulting in decreased production of IFN-γ, TNF-α and other cytokines that are important in controlling both pathogens [14–18].

Several interventions have been implemented to address the syndemics of HIV-1 and TB. These include routine HIV testing among TB patients, increased coverage of co-trimoxazole and isoniazid preventive therapy in HIV-1-infected TB patients and earlier diagnosis of HIV and initiation of antiretroviral therapy (ART) [2]. The increased accessibility of ART has significantly improved the clinical outcome of HIV-1 infected patients and reduces the TB risk by 58–80 % [19]. The positive effects of ART have been described as triphasic, starting with an early and rapid rise of CD45RO^+^ memory T cells that redistribute from lymphoid tissues into the plasma within the first month of therapy, followed by a reduction in T cell activation and improved CD4^+^ reactivity to recall HIV-1 antigens, and finally the slower replenishment of the CD45RA^+^ naïve T cells [20]. In a longitudinal study of co-infected patients sensitized by *M. tuberculosis*, ART led to an increase in central memory (CD27^+^CD45RA^−^ and CD27^+^CCR5^−^CD4^+^) T cells by 12 weeks post-treatment, followed by an increase in naïve (CD27^+^CD45RA^+^) T cells at 36 weeks [21]. A separate longitudinal study confirmed that the polyfunctional effector memory (CD27^−^CD45RO^+^) and terminally differentiated memory (CD27^−^CD45RO^−^) CD4^+^ T cells responsive to mycobacterial purified protein derivative (PPD) were restored 12 months post-ART [22].

## Immune reconstitution inflammatory syndrome

Although ART has positive effects through suppression of HIV-1 viral load and restoring CD4^+^ T cell numbers, this rapid restoration of the immune system can also lead to an undesirable complication known as immune reconstitution inflammatory syndrome (IRIS; also known as immune restoration disease, IRD). IRIS is characterized by a transient but sometimes severe local and systemic inflammatory response directed against a known condition (e.g., opportunistic pathogens or autoimmune diseases) in HIV-1 infected patients shortly after ART initiation. It was first reported in 1992, where HIV-1 co-infected patients developed *Mycobacterium avium*-complex (MAC) disease with severe lymphadenopathy and high fever following commencement of zidovudine monotherapy [23]. IRIS was subsequently documented to be associated with many different pathogens, including cytomegalovirus (CMV), hepatitis B and C viruses, *Cryptococcus*, and *M. tuberculosis*, as well as in cancer (Kaposi’s sarcoma and non-Hodgkin’s lymphoma) and autoimmune diseases [24].

In a meta-analysis on 54 cohort studies, Muller et al. reviewed the incidence and mortality of IRIS associated with different health conditions [25]. CMV-IRIS was found to have the highest incidence (pooled estimates 37.7 %), while cryptococcal meningitis-IRIS had the highest mortality (pooled estimates 20.8 %). More recently, a meta-analysis on 40 studies indicates the overall incidence of IRIS is ~18 % among patients with HIV-associated TB, with mortality attributable to TB-IRIS of ~2 % [26]. Patients with TB meningitis (TBM), however, are at high risk of developing IRIS (47 %), with a mortality rate of up to 30 % following development of IRIS [27–29].

### Clinical definition of TB-IRIS

There is no diagnostic test for TB-IRIS and diagnosis therefore relies on medical history, laboratory data, and clinical presentation. Prior to 2006, a number of TB-IRIS case definitions existed [30–34], but the lack of consensus between these definitions hindered clinical management and research on IRIS. In response to the increase in incidence of this complication in resource-limited settings, over 100 researchers gathered to agree consensus case definitions for ART-associated TB and TB-IRIS [35], which have several times been re-investigated prospectively and validated [36–39].

There are two forms of TB-IRIS that share clinical features but are distinct in their temporal relationships between tuberculosis diagnosis and treatment and the initiation of ART: paradoxical and unmasking. In paradoxical TB-IRIS, HIV-1-patients are diagnosed with TB prior to ART commencement and TB pathology has stabilized or improved during antitubercular therapy. Following ART initiation, patients experience new, recurrent or worsening features of TB, such as lymph node swelling and abscess formation, serositis, and radiographic deterioration [35]. In unmasking TB-IRIS, patients with previously undiagnosed and untreated TB present after initiation of ART and with often marked inflammatory features of TB [35]. Exclusion criteria for both types of TB-IRIS include exacerbation of TB due to drug-resistant *M. tuberculosis*, presence of other opportunistic infections, poor drug adherence, or adverse drug reactions.

### Risk factors for TB-IRIS

Although the underlying mechanisms of TB-IRIS pathogenesis are incompletely elucidated, A few clinical risk factors have been related to developing TB-IRIS. Firstly, patients with low CD4^+^ T cell counts at the time of ART initiation, followed by a rapid increase of CD4 counts post-ART, are more likely to develop TB-IRIS [40–43]. This contributes to an exaggerated T cell response directed to *M. tuberculosis* along with overproduction of both pro- and anti-inflammatory cytokines [44]. The second risk factor is a short interval between starting antitubercular therapy and ART [45–47]. The CD4 counts should be taken into consideration when deciding the optimal time to initiate ART. For patients with low CD4^+^ T cell counts (<50 cells/μl), the benefits of early initiation of ART in reducing mortality and opportunistic infections outweigh the risk of IRIS and therefore ART should not be delayed more than 2 weeks. For those with CD4 counts >50 cells/μl, ART should be commenced between 2 and 12 weeks after starting TB treatment. Furthermore, dissemination of TB infection to extrapulmonary organs appears to increase the risk of TB-IRIS by up to eight-fold, probably due to higher bacterial burden in such cases [48, 49]. TBM is the most severe form of extrapulmonary TB and in one series accounted for 12 % of all TB-IRIS cases [27]. Finally, high HIV-1 viral load is another risk factor for TB-IRIS [26] and *M. tuberculosis* culture positivity in the cerebrospinal fluid (CSF) is a risk factor for TBM-IRIS [27].

### Management

The inflammatory response associated with TB-IRIS can be transient and may resolve without specific intervention. However, in many cases there is marked inflammation that, at worst, may result in death. Anecdotal reports of glucocorticoids to reduce inflammation in TB-IRIS [50–52] encouraged a randomized placebo-controlled trial to evaluate TB-IRIS treatment with prednisone [53]. Patients with paradoxical TB-IRIS who received a 4-week course of prednisone (1.5 mg/kg per day for 2 weeks then 0.75 mg/kg per day for 2 weeks) were found to have reduced need for hospitalization, quicker resolution of symptoms and a better quality of life without excess of severe adverse events, compared to those who received placebo [53]. The beneficial effects of prednisone in reducing the inflammatory reactions in TB-IRIS were associated with more rapid resolution of the elevated C-reactive protein (CRP) and suppression of pro-inflammatory cytokines of innate immune origin [54]. Nevertheless, prednisone should be prescribed and monitored with caution in patients with advanced HIV-1 infection as (albeit outside the context of widespread ART) it associates in some studies with an increase in the risk of HIV-1 related malignancy (such as Kaposi’s sarcoma) and recurrent herpes zoster [55, 56]. Furthermore, as TB-IRIS may occur in patients with drug-resistant *M. tuberculosis,* prednisone therapy should be used with care pending satisfactory optimization of antimicrobial therapy [57].

In addition to glucocorticoids, other anti-inflammatory agents have also been used to treat TB-IRIS or IRIS associated with other pathogens in isolated cases (Table 1). Non-steroidal anti-inflammatory drugs (NSAIDs) have been used to relieve symptoms in non-severe cases of paradoxical TB-IRIS, MAC-IRIS, and cryptococcal-IRIS with lymphadenitis with favorable results [58]. Thalidomide is an immunomodulatory drug with use in cancer and inflammatory diseases. It has been used to treat patients with intractable tuberculomas [59, 60] and alleviated symptoms in steroid-refractory TB-IRIS, TB- and cryptococcal-lymphadenitis IRIS, as well as in pediatric neurological TB-IRIS [61–64]. Leukotriene antagonists (e.g., montelukast and zileuton) are used in treatment in asthma and most recently have been proposed as potential host-directed therapy in active TB [65]. Montelukast has been used to treat two cases of steroid-refractory IRIS associated with TB and syphilis and also in a patient with urticarial IRIS vasculitis, with rapid clinical responses [66, 67]. Other drugs such as pentoxifylline and hydroxychloroquine had also been used to treat isolated cases of IRIS associated with different pathogens with some reported benefits [68–71]. Nevertheless, these therapies lack clinical trial data to assess their effectiveness and potential adverse effects in treating TB-IRIS and further rigorous evaluations are required.

**Table 1:** Drugs used to treat TB-IRIS

Drug/class of drugs	Mechanism of action	Potential side effects
Corticosteroids	At gene level, activate the transcription of anti-inflammatory mediators and inhibit transcription of pro-inflammatory genes (e.g., cyclooxygenase and cytokines). At cellular level, reduce the production of nitric oxide and inhibits TCR signaling, thereby reducing cell migration, proliferation and effector function	Increased risk of Kaposi’s sarcoma, herpes simplex and zoster flare. General side effects include increased risk of infections, hypertension, diabetes, osteoporosis, ulcers and mental health problems
NSAIDs	Relieve pain and reduce inflammation by inhibiting cyclooxygenase-1 and −2, thereby reducing the synthesis of inflammation mediator prostaglandins	Increased risk of gastrointestinal problems such as ulcers; not recommended in patients with a history of renal or liver disease
Thalidomide	Modulates the production of cytokines and inflammatory mediators; also stimulates T cells and modulates NK cell cytotoxicity	Peripheral neuropathy, somnolence, hepatotoxicity, teratogenicity, skin reactions, constipation, tremor, mood changes and headache
Leukotriene receptor antagonists	Blocks pro-inflammatory leukotrienes by inhibiting the 5-lipoxygenase pathway or by antagonizing cysteinyl-leukotriene type 1 receptors; inhibit leukocytes trafficking to the sites of antigen stimulation	Skin reaction, sinus pain, tremors, mood changes, gastrointestinal problems; may also interact with rifampicin and antiretroviral drugs
Pentoxifylline	A non-selective adenosine receptor antagonist and also non-selectively inhibits phosphodiesterase, resulting in an increase in cAMP activity and reduced inflammation. In addition, it improves erthyocytes deformability, decreases blood viscosity and inhibits neutrophil adhesion and activation	Hemorrhage, gastrointestinal problems, nausea, dizziness, blurred vision, flushing and chest pain
Hydroxychloroquine	Blocks activation through TLR and interferes with MHC-II processing; reduce synthesis of pro-inflammatory cytokines	Blurred vision, somnolence, gastrointestinal problems, skin rash and lost of appetite

Drugs with reported use in treating TB-IRIS, their mechanism of actions and side effects are listed

Finally, TNF-α inhibitors (infliximab, etanercept, adalimumab ,or certolizumab pegol) are effective in treating inflammation in rheumatoid arthritis, spondyloarthropathies, and Crohn’s disease. While TNF-α inhibitors are to be prescribed with caution in patients with latent TB due to increased risk of reactivation, in one case withdrawal of adalimumab led to a life-threatening paradoxical TB reaction and the symptoms resolved when the patient was restarted on adalimumab [72]. In another case study of steroid-refractory TBM, the patient improved upon adalimumab therapy [73]. Similar observations have been reported with another TNF-α inhibitor infliximab, where the patient suffered a debilitating TB paradoxical reaction unresponsive to corticosteroids and cyclophosphamide yet showed a favorable response following three doses of infliximab given at monthly intervals [74]. In a single case study with HIV-related inflammatory cerebral cryptococcoma, patient had improved disease symptoms following treatment with adalimumab along with isoniazid and pyridoxine (to prevent tuberculosis reactivation) [75]. Since elevated levels of TNF-α are associated with TB-IRIS [76, 77], TNF-α inhibitors may offer potential benefits as treatment for TB-IRIS.

## Inflammatory reactions in TB-IRIS

TB-IRIS results from excessive inflammatory reactions against *M. tuberculosis* antigen driven by ART-induced reconstitution of the immune system [44, 78]. Numerous reports have described aspects of the immune responses observed in TB-IRIS, which we will discuss in detail in the following sections.

### CD4^+^ T cells and TCRγδ T cells

Paradoxical TB-IRIS was initially ascribed to acute expansion of the mycobacteria-specific Th1 response following ART commencement [44]. The first patient cohort study on TB-IRIS consisted of 19 HIV-TB patients, 7 of whom developed IRIS during the first month of ART. Compared to the non-IRIS patients at 3 months, those with TB-IRIS were found to have significantly higher number of IFN-γ-producing Th1 cells specific to mycobacterial PPD, but not to *M. tuberculosis*-specific ESAT antigen or CMV [44]. Furthermore, peripheral blood mononuclear cells (PBMC) from a subset of four patients (three TB-IRIS and one non-IRIS) were stimulated with PPD antigen and those with TB-IRIS secreted significantly higher concentrations of Th1 (IL-2, IL-12, IFN-γ, and IP-10) and other (TNF-α, IL-1β, IL-6, IL-10, RANTES, and MCP-1) cytokines and chemokines, while Th2 cytokines (IL-4, IL-5, IL-13, and IL15) were not detected [44]. The presence of *M. tuberculosis* antigen-specific Th1 expansions in TB-IRIS was confirmed in a separate study of 95 HIV-TB patients (35 TB-IRIS, 29 non-IRIS and 31 ART-naïve) [79]. However, not all cases of TB-IRIS were characterized by Th1 expansions and conversely expansions occurred longitudinally during ART in many patients who did not develop TB-IRIS. The proportion of HLA-DR^+^CD71^+^ CD4^+^ and CD8^+^ T cells was similar in TB-IRIS and non-IRIS controls [79]. Two additional studies by Tieu et al. [80] and Elliott et al. [81] utilized interferon-gamma release assay (IGRA) and did not find any difference in IFN-γ response in whole blood between patients with paradoxical TB-IRIS and the non-IRIS controls 12 weeks post-ART, although the latter study observed an increase in IFN-γ response to PPD 24 weeks post-ART.

Since IRIS commonly occurs in patients who are severely lymphopenic following ART initiation, Sereti and colleagues proposed that the syndrome is contributed by lymphopenia-induced T cell homeostatic mechanisms, and an imbalance in the regulatory mechanisms occurs irrespective of antigen stimulus [82]. In a longitudinal study of 45 HIV-1-infected patients commencing ART, 16 developed IRIS associated with different etiological causes and were found to have a highly activated, predominantly PD-1^+^ HLA-DR^+^ and Ki67^+^ CD4^+^ T cell phenotype prior to and during IRIS, compared to the non-IRIS controls [82]. Moreover, these IRIS patients also had a Th1/Th17-skewed cytokine profile upon polyclonal stimulation and increased PD-1 and Ki67 expression in regulatory T cells (Treg), suggesting increased activation of effector T cells due to antigenic exposure. Furthermore, TB-IRIS patients were found to have a significantly higher serum concentration of IL-7 and sCD25 compared to the non-IRIS controls [82, 83], further indicating that TB-IRIS is associated with exaggerated T cell activation and proliferation. In a follow-up study, it was noted that patients with IRIS related to different pathogens did not have a generalized T cell dysfunction and the dysregulated CD4^+^ T cell response represented an upsurge of pre-existing responses that were both polyfunctional and targeted exclusively to the opportunistic pathogen, not to HIV-1 itself [84].

In addition to Th1, other T cell subsets have also been investigated for their potential contribution to TB-IRIS. As mentioned above, Foxp3^+^ CD4^+^ T cells (considered as Treg) cells are critical in maintaining T cell homeostasis [85]. In mice infected with TB, Treg cells expanded and accumulated at the site of infection and contribute to suppression of Th1-type immune responses [86]. Increased circulating Treg frequency was also reported in TB patients, in which blood CD4^+^ T cells were found to have increased cell surface expression of CD25^high^ and increased mRNA expression of Foxp3 [87]. Several studies have investigated whether a dysregulated Treg response may contribute to TB-IRIS and reported conflicting results. Two of these studies found an expansion of Foxp3^+^ Tregs in *M. avium* and CMV-associated IRIS, although the ability of these cells to secrete IL-10 appeared to be compromised in vitro [88, 89]. Conversely Meintjes et al. reported an overall low level of Foxp3^+^ T cells among PBMC, with no difference in the percentage of these cells in TB-IRIS and non-IRIS patients [79]. Similar observations were also reported by Antonelli et al.: neither the number nor the frequency of Treg differed when comparing IRIS to non-IRIS patients [82].

The role of TCRγδ T cells in TB-IRIS has also been explored. TCRγδ T cells are potent producers of IFN-γ and TNF-α, possess cytotoxic capacity and release granule-associated perforin and granzyme B to lyse infected cells upon antigen recognition [90]. They are activated in response to *M. tuberculosis* early after infection and may reduce intracellular bacterial growth [91]. The Vδ2^+^TCRγδ^+^ T cell subset are ascribed a role in adaptive immunity to mycobacterial infection [92, 93] and their numbers are restored following successful antitubercular treatment in HIV-1 infected persons [94]. Patients co-infected with HIV-1 and *M. tuberculosis*, however, have significantly reduced TCRγδ T cell responses [91]. Compared to non-IRIS patients, those with paradoxical TB-IRIS were found to have significantly higher number of killer immunoglobulin receptor (KIR)^−^Vδ2^+^TCRγδ^+^ T cells but a lower number of KIR^+^TCRγδ^+^ T cells at all timepoints including baseline [95]. This observation indicates independent regulation of different TCRγδ T cell subsets during IRIS development, with the suggestion that the KIR^−^Vδ2^+^TCRγδ^+^ subset amplifies the dysregulated immune response to *M. tuberculosis*, whereas the inhibitory KIR^+^Vδ2^+^TCRγδ^+^ subset was suppressed.

Together, these data demonstrate a role for CD4^+^ T cells and other T cell subsets in the dysregulated inflammatory response in TB-IRIS. Nevertheless, as some TB-IRIS cases are not associated with T cell expansion, it remains conjectural whether the T cell response is the cause, rather than the consequence, of the syndrome.

### CD8^+^ T cells

Compared to CD4^+^ T cells, there have been very limited studies on the role of CD8^+^ T cells in TB-IRIS pathogenesis. Two studies showed that the numbers and frequency of CD8^+^ T cells did not differ between TB-IRIS and non-IRIS patients prior to ART and during IRIS [35, 96]. In contrast, Reyes-Teran and colleagues reported that increased frequency of circulating CD8^+^ T cells is a risk factor for developing IRIS associated with *M. tuberculosis* and *M. avium* [97]. Furthermore, the authors reported an expansion of the naïve CD8^+^ subpopulation, as well as the CD38^+^ HLA-DR^+^ CD8^+^ T cells, during TB-IRIS episode [97]. No further phenotyping of the CD8+ T cells in the study was performed, thus the precise subpopulation associated with TB-IRIS development was not defined. Furthermore, the functionality of CD8^+^ T cells has not been investigated in the context of TB-IRIS and their contribution to the syndrome remains unclear.

### Soluble mediators

A feature of TB-IRIS is increased production of both pro- and anti-inflammatory cytokines, which is in turn influenced by the antigen load in patients [98, 99]. In the study by Tadokera et al., PBMC from paradoxical TB-IRIS patients stimulated with heat-killed *M. tuberculosis* secreted significantly higher concentrations of IL-1β, IL-2, IL-6, IL-8, IL-10, IL-12p40, IFN-γ, GM-CSF, and TNF-α, compared to those from the non-IRIS controls [77]. Higher concentrations of IL-6, IL-8, IL-10, IL-12p40, IFN-γ, and TNF-α were also detected in the serum of the same TB-IRIS patients. Similar observations have been reported by others [100–102]. In a study comparing TB-IRIS patients randomized to prednisone or placebo, the serum concentration of IL-6, IL-10, IL-12p40, IFN-γ, IP-10, and TNF-α decreased during 4 weeks of prednisone therapy, but not in the placebo group [54], further implying a pathological role for hypercytokinemia in TB-IRIS. IL-6 has been suggested to be a major pathological mediator in TB-IRIS as a higher level of plasma IL-6 and CRP at baseline was associated with subsequent development of TB-IRIS [103]. Barber et al. assessed whether inhibition of IL-6 can reduce pathology in a murine model with *M. avium*-associated IRIS (see “Animal model” section) and showed that neutralization of IL-6 with a monoclonal antibody reduced CRP levels, alleviated disease pathology and extended survival [104]. Most recently, Ravimohan et al. reported that inflammatory markers increased rapidly in HIV-TB patients with early deaths and in patients who developed TB-IRIS, but the two groups have differential recovery of the adaptive immune system [76]. Patients with early mortality were found to have increased pre-ART plasma concentrations of IL-6, IL-10, TNF-α, MCP-1, and eotaxin, but these increases were not accompanied by early recovery of CD4^+^ T cells. In contrast, the significant increase in the plasma concentrations of IL-6 and TNF-α following ART initiation paralleled a marked increase in CD4 counts in TB-IRIS patients. Together, the CD4 counts prior to and following ART, along with the concentrations of inflammatory markers such as IL-6 and TNF-α might have potential use as biomarkers to assist TB-IRIS diagnosis.

Furthermore, plasma or serum concentrations of IL-17 and IL-22 have also been reported to be higher in both paradoxical and unmasking TB-IRIS [100, 105]. Both IL-17 and IL-22 arise from distinct lineages of T cells and are critical in bridging innate and adaptive immunity in host defense against pathogens at mucosal sites (reviewed in [106]). More recently, elevation of plasma IL-18 at baseline (pre-ART) and during TB-IRIS has also been reported [107, 108]. In addition to the excessive release of both pro- and anti-inflammatory cytokines, IP-10 and CCL4 chemokines were also found to be elevated in the plasma of TB-IRIS patients longitudinally over the course of ART [107, 109]. Similar findings were reported in patients with TBM-IRIS, where the concentrations of an array of chemokines (CXCL1-3, CCL2/3/4, and IP-10) were all significantly higher in the CSF of TBM-IRIS patients compared to non-IRIS TBM patients [110]. The induction of these chemokines suggests an increased chemoattraction of monocytes/macrophages and other immune cells to the site of inflammation, thereby contributing to the pathogenesis of TB-IRIS.

Other inflammatory mediators have also been reported to be elevated in TB-IRIS.

Matrix metalloproteinases (MMPs) are zinc-dependent endopeptidases involved in tissue repair, remodeling and modulation of the immune response [111, 112]. The concentration of MMP-1 was significantly higher in the lung of TB patients with or without HIV-1 infection [113]. In the context of TB-IRIS, PBMC from paradoxical TB-IRIS patients were found to have significantly higher mRNA expression and protein secretion of MMP-1, -3, -7 and -10 than in non-IRIS controls [114]. Following prednisone therapy, the serum concentration of MMP-7 showed a modest reduction, suggesting a specific contribution to pathogenesis. Similarly, in the CSF of TBM-IRIS patients, the concentration of MMP-9 was significantly higher compared to non-IRIS TBM patients both pre-ART and post-ART [110]. However, the CSF concentration of MMP-9 did not decrease following antitubercular and corticosteroid therapy and continued to rise following ART initiation. This, together with the poor clinical outcomes, suggests that more potent and specific therapy may be needed for the management of TBM-IRIS.

Finally, deficiency of vitamin D_3_ is associated with active TB disease [115]. Adjunctive vitamin D_3_ supplementation can help resolve TB pathology by suppressing antigen-stimulated pro-inflammatory response and inhibiting secretion of MMP-1, -7, -9 and -10 [116, 117]. While deficient plasma levels of vitamin D_3_ did not predict the development of TB-IRIS [118], serum concentrations of IL-1β, IL-6, and IL-8 were significantly higher at both baseline and during TB-IRIS in vitamin D_3_-deficient patients who experienced the syndrome [109].

In patients with TBM, concentrations of inflammatory mediators were significantly higher in the CSF than in blood regardless of IRIS status [110]. Individuals who eventually developed TBM-IRIS had elevated CSF concentrations of an array of cytokines, chemokines, MMPs, as well as neutrophil-associated mediators (e.g., S100A8/A9) at both baseline and the time of symptom presentation [110]. Compared to CSF, representative of the neurological compartment, only subtle differences in the concentrations of these analytes were detected in the corresponding blood samples between TBM-IRIS and non-IRIS. Further studies conducted with materials from IRIS disease sites are likely to be informative.

The potential genetic influence in IRIS predisposition has been investigated in two studies by Price and colleagues. While the small sample size rendered these studies underpowered and inconclusive, they nevertheless provided some pointers that genetic factors might play a role in TB-IRIS. In a study that included nine patients with MAC-associated IRIS and two with TB-IRIS, the frequency of the IL6-174*C polymorphism (36 %) was significantly lower than in the non-IRIS controls (61–71 %) [119]. Furthermore, none of the IRIS patients carried the TNFA-308*2 polymorphism, while the frequency was 23–52 % in the non-IRIS group. In a cohort study with 17 Cambodian and 19 Indian patients, TB-IRIS was associated with higher frequencies of TNFA-1031*T and SLC11A1 D543*G in Cambodian patients, while higher frequencies of IL18-607*G and VDR Fokl(F/f)*T were observed in Indian patients [120]. Larger-scale studies would be required to determine with confidence if specific polymorphisms relate to the risk of TB-IRIS, although recruitment to such a study might be difficult.

### B cell response

TB-IRIS is regarded as a cell-mediated disease and little is known about the role played by humoral immunity. Simmoney et al. examined longitudinal antibody responses in a cohort of 24 HIV-TB co-infected patients starting on ART by measuring circulating free and immune-complexed antibodies against *M. tuberculosis* antigens (ManLAM, ESAT-6/CFP10, and PGL-Tb1) [121]. Compared to non-IRIS patients, those with TB-IRIS had a significantly lower level of anti-PGL-Tb1 antibody prior to the episode regardless of CD4 counts or presence of complexed antibodies. There was no difference in the antibody levels against ManLAM or ESAT-6/CFP-10 between TB-IRIS and non-IRIS. A similar result was also reported in another study where the levels of anti-PPD, anti-ManLAM, and anti-38-kDa antigen antibodies did not differ between TB-IRIS and non-IRIS patients prior to ART, or at the time of IRIS [122]. Furthermore, the lineage of *M. tuberculosis* did not appear to be associated with development of TB-IRIS [121], although this interesting issue is not resolved with certainty.

More recently, a regulatory role for B cells has been described in TB granulomas in primates, which is mediated via secretion of IL-10 and *M. tuberculosis*-specific antibodies [123]. Antibodies contribute to immune defense through three mechanisms: neutralization, activation of the complement system, or opsonization. In opsonization, phagocytes expressing Fcγ receptors recognize antibody-bound antigens (e.g. *M. tuberculosis*) and take up the complex into the phagosomes for elimination. Increased expression of Fcγ receptors (FCGR1/2/3) has been reported in almost all transcriptional studies on active TB disease [124] and was also identified in microarray profiling of TB-IRIS patients during the IRIS episode [125]. Together, these data suggest that humoral immunity may also contribute to TB-IRIS pathogenesis, although the precise mechanisms remain poorly defined.

### Innate immune response

The role of innate immunity in TB-IRIS pathogenesis has attracted increased attention in recent years. Myeloid cells are the main cells targeted by *M. tuberculosis* and their pivotal role in antimicrobial defense has been extensively described. Colebunders and colleagues speculated that macrophages in HIV-1 patients with advanced disease, particularly those co-infected with *M. tuberculosis*, are inappropriately activated [126]. Previous studies have reported that stimulation of HIV-1 gp120 alone is sufficient to induce dysfunction and aberrant gene expression in monocytes or monocytes-derived macrophages [127–129]. During ART, these immunosuppressive phenotypes are reversed and the functional recovery could result in excessive activation of the macrophages by *M. tuberculosis* antigens [126], subsequently contributing to development of IRIS. Lawn et al. later reported a fatal case of unmasking TB-IRIS, in which CD68^+^ macrophages were identified as the predominant inflammatory cells in postmortem staining of lung tissue sections [130].

Human monocytes can be categorized into three different subsets: classical CD14^++^CD16^−^ and non-classical CD14^+^CD16^+^ and CD14^dim^CD16^+^. The roles played by these subsets in TB-IRIS have recently been explored. Peripheral CD14^+^CD16^+^ monocytes were isolated from TB-IRIS and non-IRIS patients and transcriptional profiling was performed. Genes associated with the complement system and pattern recognition receptors were found to be differentially abundant in TB-IRIS [131, 132]. The authors further investigated the role of complement in TB-IRIS and reported significantly higher levels of C1q and C1-inhibitor of the classical pathway at baseline and an imbalance in their ratio during TB-IRIS onset at 2 weeks post-ART. Activation of the complement system is known to trigger opsonization and killing of pathogens as well as recruitment of inflammatory cells [133]. In another study that examined TB-IRIS patients from South India and South Africa, TB-IRIS was associated with increased plasma concentrations of sCD14, sCD163, and soluble tissue factor, all of which are markers of monocyte activation [134]. Furthermore, patients with TB-IRIS lacked CD14^dim^CD16^+^ monocytes while the frequency of CD14^++^CD16^−^ monocytes were significantly higher than in non-IRIS patients. CD14^++^CD16^−^ monocytes have high expression of the activation marker CD163 and were found to be closely associated with plasma levels of systemic pro-inflammatory markers CRP, IL-6, TNF-α, and soluble tissue factor (CD142) prior to ART initiation and during TB-IRIS [134]. Together, these data point towards a pathogenic role for both classic and non-classical monocytes in TB-IRIS and the mechanisms mediated by these cells should be investigated further.

In addition to macrophages/monocytes, natural killer (NK) cells and invariant natural killer T (iNKT) cells may also contribute to TB-IRIS pathogenesis. Activated NK cells can protect against *M. tuberculosis* either directly by lysing infected monocytes and antigen-specific Treg [135, 136], or indirectly by restoring the frequency of *M. tuberculosis*-specific CD8^+^IFN-γ^+^ T cells [137]. Patients with unmasking TB-IRIS were found to have increased NK cell activation and plasma concentrations of CRP and IL-8, compared to non-IRIS or HIV-1-monoinfected controls [138]. In another cohort of 128 co-infected patients from Cambodia, baseline NK cell degranulation capacity was significantly higher in those who eventually developed paradoxical TB-IRIS, but this difference was abrogated following ART initiation, possibly due to regulatory feedback mechanisms or the internalization of NK receptors following ligand binding [139]. Finally, microarray analysis performed on *M. tuberculosis*-stimulated PBMC isolated from patients identified an overabundance of granzyme B and perforin transcripts in TB-IRIS, validated at the protein level [96]. The increased secretion of perforin appeared to be contributed by an increased number of CD3^+^Vα24^+^ iNKT cells. Activation of CD1d-restricted iNKT cells has previously been shown to protect mice from *M. tuberculosis* infection via a yet unknown mechanism [140]. Further studies are needed to address the role of cytotoxic cells in TB-IRIS.

Neutrophils also appear to have a significant role in the pathogenesis of the highly compartmentalized TBM-IRIS. Active TB disease in HIV-1 uninfected persons is characterized by a type I interferon-inducible, neutrophil-driven transcriptomic signature [141]. Mice lacking IFN1 receptor were protected from TB-induced death with reduced recruitment of neutrophils and inflammatory macrophages to the site of infection [142]. In a cohort of HIV-1-infected South African patients diagnosed with TBM, those who did not develop TBM-IRIS but who were TB culture positive in their CSF showed similar inflammatory response as the TBM-IRIS patients both at the time of TBM diagnosis and 2 weeks post-ART [88]. However, TBM-IRIS was specifically associated with elevated neutrophil counts in CSF and increased expression of the neutrophil mediators S100A8/9 [27, 110].

**Fig. 1:**
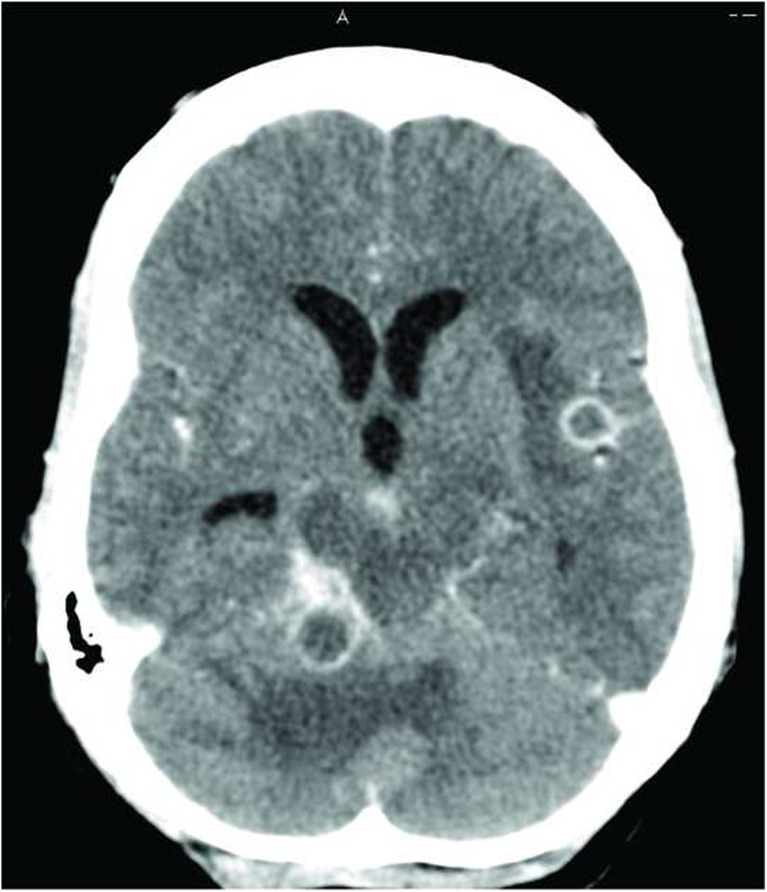
Contrast-enhanced axial computed tomography (CT) image of TBM-IRIS. CT image showing multiple ring-enhancing lesions with surrounding edema and hydrocephalus in a 41-year-old woman. The patient previously presented with TBM (CSF TB culture was positive for *M. tuberculosis* susceptible to rifampicin and isoniazid) 10 weeks prior to this presentation. At that time, she was started on TB treatment and ART was initiated 9 weeks later. One week after initiating ART, the patient developed recurrent headaches and this CT was performed 2 weeks later. The recurrent symptoms and these CT findings were ascribed to TBM-IRIS and the patient was treated with corticosteroids with symptom improvement. Image provided by Dr. Suzaan Marais

**Fig. 2:**
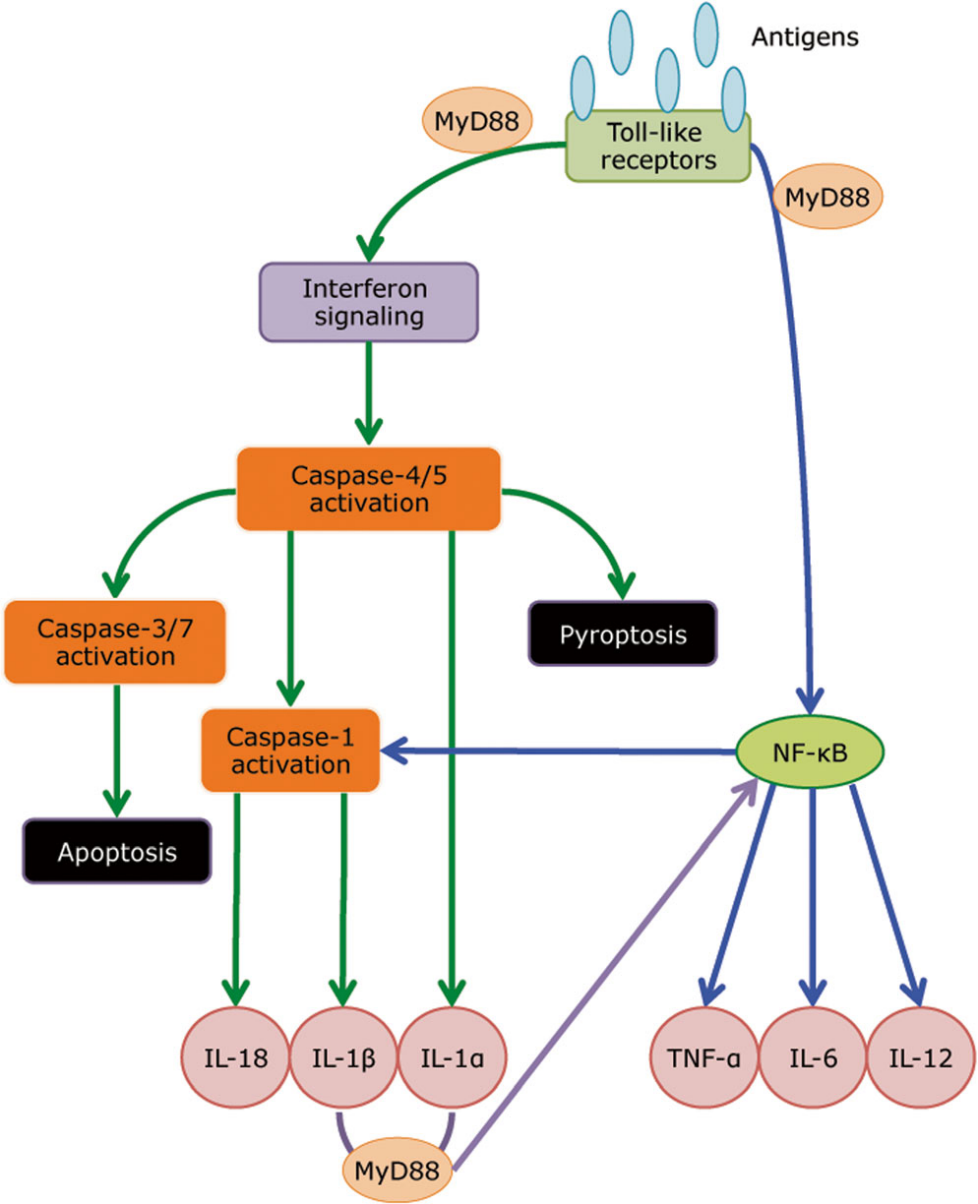
A model of innate receptor signaling in mediating TB-IRIS pathogenesis. Microarray profiling revealed that TLR signaling and inflammasome activation are critical in mediating TB-IRIS pathogenesis (Table 2) [125]. Our proposed model begins with *M. tuberculosis* antigen recognition by surface-expressing TLRs, which triggers the downstream signaling cascade with adaptor molecules such as MyD88 and IRAK4 to activate IRF7, thereby triggering the production of type I IFN. Paracrine signaling of Type I IFN to IFNAR recruits and phosphorylates STAT1/2 dimers, leading to further recruitment of IRF9 and the formation of ISGF3, thereby inducing pro-caspase-11 (caspase-4/5 in human) and AIM-2 inflammasome (caspase-1). Caspase-11 cleaves IL-1α into its mature form and can lead to pyroptosis. The noncanonical inflammasome (caspase-11) can also activate the canonical inflammasome (caspase-1), which cleaves IL-1β and IL-18 into their mature form. Alternatively, TLR signaling via MyD88 can also activate NF-κb via the TAK1/IKK complex. Activation of NF-κb triggers the production of an array of cytokines, including TNF-α, IL-6 and IL-12. In addition, NF-κb can also activates NLRP1/3 inflammasomes and subsequently leads to the production of IL-1β and IL-18

Most recently, we have employed transcriptomic profiling of whole blood to investigate potential mechanisms that underlie TB-IRIS pathogenesis. In a longitudinal study whose endpoint was the development of TB-IRIS, we closely tracked responses at various timepoints prior to and following ART commencement (within days of starting ART and up to the median time of IRIS occurrence at 2 weeks). We identified transcripts of TLR signaling and activation of inflammasomes to be prominent in the blood of TB-IRIS patients (Table 2) [125]. Both TLR2 and TLR4 have been shown to recognize *M. tuberculosis* antigen. Mice lacking TLR4 have reduced capacity to eliminate *M. tuberculosis* from the lungs and succumbed to disease sooner than wild-type controls [143]. A separate mouse study showed that in high-dose aerosol infection, TLR2 is critical in mediating innate defense against *M. tuberculosis* infection [144]. Furthermore, TLR2 was previously reported to express at higher baseline levels in myeloid dendritic cells and monocytes in patients with paradoxical and unmasking TB-IRIS, compared to non-IRIS, and its expression remained higher even at 24 weeks post-ART initiation [145]. This observation suggests that dysregulated signaling via TLR2 mediates the inflammatory reactions observed in TB-IRIS. Indeed, when we inhibited MyD88 (the downstream adaptor of TLR2) in patient’s PBMC in vitro, secretion of pro-inflammatory cytokines was markedly reduced specifically in TB-IRIS patients [125]. The production of IL-1 in TB-IRIS appeared to be dependent on inflammasome activation. In vitro inhibition of Caspase-1/4/5 in PBMC from TB-IRIS patients reduced IL-1 secretion, probably due to disruption in the cleavage from immature to mature form. Together, these data demonstrate the central role played by innate receptor signaling in mediating TB-IRIS pathogenesis and offer some mechanistic insights on the disease (Figs. 1 and 2).

**Table 2:** Canonical pathways associated with TB-IRIS

Top canonical pathways associated with TB-IRIS	*p* value	Regulation
Inflammasome activation	2.01E−04	Up
Toll-like receptor signaling	2.01E−04	Up
Endothelin-1 signaling	3.84E−04	Up
Role of pattern recognition receptors in bacteria and virus recognition	8.83E−04	Up
IL-1 signaling	8.83E−04	Up

Microarray profiling using whole blood from a cohort of TB-IRIS and TB non-IRIS patients was performed and differentially abundant transcripts associated with TB-IRIS were identified. Functional analysis by Ingenuity Pathway Analysis (IPA) indicates that these differentially abundant transcripts overrepresented innate signaling pathways, including inflammasome activation, toll-like receptor signaling and IL-1 signaling, suggesting that innate immunity plays a significant role in TB-IRIS pathogenesis

## Animal model

Since HIV-1 does not readily infect non-primates, studying mycobacteria-associated IRIS in small animal models poses a challenge. Thus far, only one murine model on IRIS has been reported using a TCRα knockout mouse chronically infected *M. avium* infection [146]. To recapitulate the CD4^+^ T cell reconstitution observed in TB-IRIS patients, purified CD4^+^ T cells from naïve mice were injected into T cell-deficient *M. avium*-infected TCRα knockout mice, which led to impaired lung function, severe wasting symptoms and increased mortality within 3 weeks of T cell transfer. In contrast, transfer of CD4^+^ T cells into wild-type chronically infected mice or TCRα knockout mice prior to *M. avium* infection did not cause any disease. Unexpectedly, the nonlymphopenic OT-II mice were also found to be susceptible to developing reconstitution disease induced by transfer of *M. avium*-specific CD4^+^ T cells, indicating that the reconstitution disease did not require a lymphopenic environment. In addition, there were two- to five-fold fewer donor CD4^+^ T cells recovered from infected compared with naïve recipients. Instead of spontaneous proliferation of CD4^+^ T cells in T cell-deficient environment, the model was associated with impaired, rather than exaggerated, T cell expansion. Furthermore, both antigen recognition and secretion of IFN-γ by the grafted CD4^+^ T cells were necessary for disease induction, which correlated with the large increase in the population of CD11b^+^ myeloid cells in the lungs and blood. While this model recapitulates the CD4 restoration characteristic in IRIS, it requires a high-dose of *M. avium* infection leading to bacterial burden that is very high. Furthermore, the model does not capture the immunosuppressive phenotype of monocytes and macrophages induced by HIV-1. Several humanized mouse models have been developed for HIV-1, some of which are able to support persistent viral infection and gradual CD4^+^ T cells decline that mimics human infection [147]. Non-human primates which share close physiology to humans have also been extensively used to study HIV-1 and TB infection, but not yet applied to TB-IRIS. While these models may better recapitulate the overall immune aspects of TB-IRIS, the high cost of performing such studies is a deterrent.

## Concluding remarks

Although TB-IRIS was first described almost 20 years ago with many subsequent case reports, the underlying mechanisms that mediate the disease pathology remain to be fully elucidated. There remains a lack of biomarkers to accurately diagnose and track the syndrome, particularly in resource-limited settings. The difficulty in studying the underlying mechanisms of TB-IRIS pathogenesis is further complicated by the absence of an animal model that can recapitulate different aspects of the immune defects observed in TB-IRIS, as well as challenges, both practically and ethically, to recruit appropriate controls groups: those who are infected only with HIV-1 or TB, treated and untreated with ART and antitubercular drugs and healthy controls. While a set of consensus guidelines adopted in recent years has helped to standardize TB-IRIS diagnosis and treatment for systematic comparison, our understanding on the pathogenesis remains incomplete. Finally, although corticosteroids such as prednisone can alleviate the symptoms and accelerate clinical improvement, they also have undesirable side effects and drugs targeting specific pathological pathways should be explored. Our group and others have recently utilized transcriptomic approaches to study the underlying mechanisms mediating TB-IRIS and the results from these studies offered insights on some of the immunological pathways involved that may guide future directions for host-directed therapy.
